# Internalizing–Externalizing Comorbidity and Impaired Functioning in Children

**DOI:** 10.3390/children9101547

**Published:** 2022-10-12

**Authors:** Megan Dol, Madeline Reed, Mark A. Ferro

**Affiliations:** School of Public Health Sciences, University of Waterloo, 200 University Avenue West, Waterloo, ON N2L 3G1, Canada

**Keywords:** adolescent, child, disability, psychiatric disorder

## Abstract

Background: The comorbidity of mental illnesses is common in child and adolescent psychiatry. Children with internalizing–externalizing comorbidity often experience worse health outcomes compared to children with a single diagnosis. Greater knowledge of functioning among children with internalizing–externalizing comorbidity can help improve mental health care. Objective: The objective of this exploratory study was to examine whether internalizing–externalizing comorbidity was associated with impaired functioning in children currently receiving mental health services. Methods: The data came from a cross-sectional clinical sample of 100 children aged 4–17 with mental illness and their parents recruited from an academic pediatric hospital. The current mental illnesses in children were measured using the Mini International Neuropsychiatric Interview for Children and Adolescents (MINI-KID), and the level of functioning was measured using the World Health Organization Disability Assessment Schedule (WHODAS) 2.0. Linear regression was used to estimate the association between internalizing–externalizing comorbidity and level of functioning, adjusting for demographic, psychosocial, and geographic covariates. Results: Internalizing–externalizing comorbidity in children was associated with worse functioning compared to children with strictly internalizing comorbidities, β = 0.32 (*p* = 0.041). Among covariates, parent’s psychological distress, β = 0.01 (*p* = 0.004), and distance to the pediatric hospital, β = 0.38 (*p* = 0.049) were associated with worse functioning in children. Conclusions: Health professionals should be mindful that children with internalizing–externalizing comorbidity may experience worsening functioning that is disruptive to daily activities and should use this information when making decisions about care. Given the exploratory nature of this study, additional research with larger and more diverse samples of children is warranted.

## 1. Background

Mental disorders are commonly categorized into two distinct groups of psychiatric disorders or symptoms—internalizing and externalizing [1]. Internalizing disorders are characterized by distress directed inwards, including mood disorders such as major depressive disorder (MDD) and anxiety disorders such as generalized anxiety disorder (GAD), separation anxiety, and phobias [2]. Externalizing disorders are characterized by distress directed toward the individual’s environment or toward others, including attention-deficit hyperactivity disorder (ADHD), oppositional defiant disorder (ODD), conduct disorder (CD), and substance use disorders [2]. Comorbidity of mental disorders is common among youth [3,4]. Research and mental illness classification schemes suggest that diagnoses are not distinct entities and most children with one mental illness meet the diagnostic criteria for another, indicating that comorbidity is the ‘rule rather than the exception’ [5].

Comorbidity of mental disorders commonly occurs within the domains of internalizing and externalizing disorders; however, comorbidity between internalizing and externalizing disorders is also prevalent [6,7,8,9,10]. For example, a study examining the network structure of internalizing and externalizing disorders found high comorbidity (15%) between anxiety disorders and MDD. Additionally, a high proportion of individuals (41.5%) with anxiety and/or MDD were found to have comorbid externalizing disorders [10].

Common risk factors that exist across mental illnesses increase the incidence of comorbidity of internalizing and externalizing illnesses [8]. Additionally, evidence suggests that shared traits underlie susceptibility to internalizing–externalizing comorbidity, and symptoms in one domain can become risk factors for symptom accumulation in the other [4]. Internalizing–externalizing comorbidity presents unique challenges, including earlier onset, greater use of mental health services and cost of service use, and worse functional outcomes compared to children with a single diagnosis [6,10,11]. Research indicates that children with internalizing–externalizing comorbidities have worse functioning compared to those with comorbid internalizing illnesses only [12,13,14].

Most of the extant literature on functional impairment among children with mental comorbidities has focused on obsessive-compulsive disorder (OCD), anxiety disorder, or attention-deficit hyperactivity disorder (ADHD) as the index condition. A study found that children with OCD and a comorbid externalizing disorder had poorer functioning in comparison to children with OCD and a comorbid anxiety disorder [14]. A study of children with ADHD and both externalizing and internalizing comorbidity demonstrated worse academic and social functioning compared to children with ADHD only [15]. Two additional studies found that children with an anxiety disorder and a comorbid externalizing disorder had lower functioning compared to anxiety only and comorbid internalizing groups [12,13]. Children with ADHD and comorbidities exhibit poor overall functioning [15], as well as deficits in domains of functioning including social [16,17,18,19,20], academic [15], and daily activities [21].

### Introduction

Important knowledge gaps remain in understanding functioning among children with comorbidities. First, a few studies examining child comorbidity have compared children with internalizing–externalizing comorbidity to those with strictly internalizing comorbidities [12,13,14]. Information on functioning is needed to inform the allocation of mental health services and evaluation of treatment outcomes [22]. Second, studies have employed population samples that may not be generalizable to high-risk clinical samples [23,24,25,26]. Third, no studies have assessed geographic/community-level factors, such as the distance to health services or residential instability (i.e., elevated rates of family or housing instability). Fourth, previous studies used outdated measures of functioning [16,22]; the current study utilized the World Health Organization Disability Assessment Schedule (WHODAS 2.0), which has replaced the Global Assessment of Functioning in the Diagnostics and Statistical Manual of Mental Disorders, fifth edition (DSM-5) as the gold standard measure of functioning [27].

This exploratory study aims to examine the extent to which internalizing–externalizing comorbidities compared to strictly internalizing comorbidities were associated with the level of functioning in a clinical sample of children. We hypothesized that after adjusting for covariates, children with internalizing–externalizing comorbidity would have poorer overall functioning.

## 2. Methods

### 2.1. Sample

The data came from a cross-sectional study of 100 children aged 4–17 years who were currently receiving inpatient or outpatient mental health services and their parents at a pediatric tertiary care centre in Ontario, Canada. Data collection occurred between October 2015 and March 2017. A description of the study has been published and relevant ethical approvals have been obtained [28]. Eight child-parent dyads did not complete the study, and four did not provide their postal code and were removed from the analysis. Children with only one internalizing illness (*n* = 8) or only externalizing comorbidities (*n* = 5) were excluded from the analysis. In this study, children with internalizing–externalizing comorbidities (*n* = 43) were compared to children with internalizing comorbidities (*n* = 32).

### 2.2. Measures

The Mini International Neuropsychiatric Interview for Children and Adolescents (MINI-KID) was used to measure mental illness among children in the past six months [29]. The following mental illnesses were assessed: internalizing (major depressive episode, generalized anxiety, separation anxiety, social phobia, and specific phobia) and externalizing (ADHD, oppositional defiant, and conduct disorder). The MINI-KID has strong psychometric properties [30,31].

The 36-item WHODAS 2.0 measured child functioning in six domains of daily living: cognition, getting around, self-care, getting along with people, life activities, and participation in society [32]. Guided by the statement “In the past 30 days, how much difficulty did you have in…”, parents responded using a 5-point Likert scale (1 = none to 5 = extreme or cannot do) [32]. Higher scores indicate worse functioning and the WHODAS 2.0 has excellent psychometric properties in this population [33,34].

Parental psychological distress focused on depression and anxiety. Depression symptoms were measured using the 20-item Center for Epidemiological Studies Depression Scale (CESD), which assessed negative and positive affect, somatic activity, and interpersonal relations over the past week [35]. Anxiety symptoms were measured using the 20 trait items from the State-Trait Anxiety Inventory (STAI), which assessed individuals’ propensity for perceived anxiety [36]. Higher scores for both scales indicate more impairment. Both the CESD and STAI have robust psychometric properties [37]. Because CESD and STAI scores were highly correlated (r = 0.73), a composite variable was computed/used.

The distance to the pediatric hospital was measured using postal codes, which were entered into Google Maps^®^ to obtain the shortest driving distance (km) while avoiding toll routes. Given the skewness of the data, the distance to the hospital was dichotomized as <50 or ≥50 km. Second, study data were linked to dissemination areas (DAs) from the 2016 Canadian Census using postal codes [38]. Data were linked at the DA level to the residential instability dimension in the 2016 Ontario Marginalization Index (ON-Marg) using a quintile scale [38].

Demographic information was collected on children and parents’ age, sex, and immigrant status. Parents’ marital status was categorized as being partnered (married/common law) or not; parent education as having completed a college/university degree or not; and household income in $30,000 increments from <$30,000 to ≥$150,000.

### 2.3. Data Analysis

Children with internalizing–externalizing comorbidities were compared to children with internalizing comorbidities using *t*-tests and chi-squared tests. Linear regression estimated the association between internalizing–externalizing comorbidities and functioning, adjusting for child age and sex, treatment setting (inpatient/outpatient), household income, parental psychological distress, distance to hospital, and residential instability. Due to the exploratory nature of the study α = 0.10. Analyses were conducted using SAS Studio Enterprise 3.81.

## 3. Results

### 3.1. Sample Characteristics

The characteristics of the sample are detailed in Table 1. Children had a mean age of 14.5 (SD = 2.2) years, 76% were female, and 4% were immigrants. Parents had a mean age of 46.2 (SD = 6.7) years, 87% were female, 14% were immigrants, 63% were partnered, 65% completed post-secondary education, and 37% reported annual household incomes of ≥$90,000 (2016 Census median household income). Twenty-three percent of participants lived ≥50 km from the hospital, and 31% resided in areas having the least marginalization (Q1 and Q2). The most common internalizing and externalizing illnesses were generalized anxiety (85%) and oppositional defiant disorder (51%), respectively. Children with internalizing–externalizing comorbidities were significantly younger (14.1 vs. 15.1 years), more likely to have social anxiety (49% vs. 22%), and more likely to live in an area with high residential instability (Q4/Q5: 42% vs. 16%).

### 3.2. Comorbidity and Level of Functioning

In a bivariate comparison, the mean WHODAS 2.0 score among children with internalizing–externalizing comorbidities (2.59, SD = 0.72) was significantly higher than the mean score among children with strictly internalizing comorbidities (2.32, SD = 0.55; t = −1.72, *p* = 0.089). Internalizing–externalizing comorbidity was significantly associated with worse functioning while adjusting for covariates [β = 0.32 (0.16), *p* = 0.041; Table 2]. Elevated parental psychological distress was associated with worse child functioning [β = 0.01 (0.01), *p* = 0.004]. Travel distance to the hospital was associated with poorer child functioning [β = 0.38 (0.19), *p* = 0.049].

## 4. Discussion

In this exploratory study, the level of functioning was poorer among those with internalizing–externalizing comorbidity compared to those with strictly internalizing comorbidities. This finding is consistent with previous reports in other child populations [12,13,14]. More research with larger and more diverse samples is needed.

Children with internalizing–externalizing comorbidity may experience more pervasive effects across different domains of functioning, resulting in worse overall functioning. Internalizing illnesses are associated with poorer functioning in social and self-esteem domains [39,40,41]. Alternatively, worse academic outcomes and family functioning are related to externalizing symptoms [42,43,44]. Future studies should investigate which domains of functioning are most affected by specific comorbidities to inform treatment decision-making. A better understanding of these internalizing–externalizing comorbidities can inform their course, treatment, and prevention—information critical for health professionals to appropriately assess, treat, and manage these complex disorders [45].

Evidence suggests that worse parental psychopathology is associated with poorer child functioning, possibly through parenting and family mechanisms [46,47]. Our findings were consistent and support recommendations for family-centred care for children and youth that address family needs in addition to improving child health [48]. Addressing the family is important in the context of health and illness [49] as the family is central in a child’s life, and therefore, should be a focus of care [50]. Child and family well-being improves when health services support the family in meeting the child’s needs [49]. Parents of children in inpatient psychiatric care also require care as well, as hospitalization of a child is stressful for the family [51]. As parental psychological distress and child functioning were both parent-reported in this study, it is possible that the association is spurious [52], and further research is needed to determine whether it is a function of informant bias or a true association [53].

The greater distance travelled to the pediatric hospital was related to poorer child functioning. Although no previous research has assessed this relationship, a ‘distance decay effect’ has been reported, such that greater distance from mental health care is related to decreased use of such services, particularly outpatient care [54,55,56]. Barriers to accessing services such as travelling long distances can contribute to increased emergency department use, which is associated with worse health outcomes [57,58]. It is important for healthcare practitioners to ensure that all care visits are productive and effective to benefit children who are receiving care. Residential instability was not associated with child functioning. There is an absence of literature on this topic; thus, speculatively, it is possible this is due to universal access to care in the Canadian context.

The sample in this study included a higher proportion of female participants. Our data showed that the proportion of ODD is higher among females, which contradicts previous research and indicates that ODD is found more in males [59]. Females are more likely to receive services and are also more likely to have internalizing disorders [60,61,62,63]. Of the 38 youth who screened positive for ODD in our sample, 25 were female and 13 were male. Mental illnesses are often comorbid, so a high proportion of females in this study will translate to a high percentage of females with ODD.

### Limitations

Findings should be interpreted while considering the following limitations. First, this exploratory study employed a small sample of children, and selection bias is possible. Second, while cross-sectional data prevents interference from causality, these findings are hypothesis-generating. Relatedly, potential mediating or moderating effects could not be examined. Third, parent-reported data were analyzed as not all children were age-eligible to complete the WHODAS 2.0. While the disability construct is interpreted similarly, the overall agreement is relatively low [64]. Thus, the reliance on just a parent’s report can introduce same-source bias and may not accurately reflect child perspectives.

## 5. Conclusions

Findings from this exploratory study showed that after adjusting for child, family, and community factors, parents of children with internalizing–externalizing comorbidities reported worse child functioning compared to parents of children with internalizing comorbidities only. Parental psychological distress and greater distance to the pediatric hospital were also associated with poorer child functioning. These findings have implications for research and clinical practice. Though the findings are preliminary, health professionals should remain vigilant in understanding how comorbidity profiles in children may influence their functioning. Health professionals should prioritize involving families in the treatment of their children to optimize mental health outcomes. Additionally, healthcare resources allocated towards improving mental health approaches targeting family-centred care strategies could be beneficial for children and the healthcare system moving forward. We encourage more research to investigate differences in functioning among children with various comorbidity types, with attention to specific domains of functioning, to promote the best possible outcomes for children with mental illness. Further understanding of mental comorbidity in children will help to inform treatment and prevention programs—improving clinical practice to appropriately treat and manage these complex disorders. Future research assessing factors that could impact child functioning, such as family factors and geographical distance to services, is warranted and would make a valuable contribution to the field.

## Figures and Tables

**Table 1:** Characteristics of the study sample.

	Full Sample (*n* = 75)	Internalizing–Externalizing (*n* = 43)	Internalizing (*n* = 32)	*p*
**Child characteristics**				
Age (years)	14.5 (2.2)	14.0 (2.2)	15.1 (2.2)	0.039
Female	57 (76.0)	30 (69.8)	27 (84.4)	0.143
Immigrant	3 (4.0)	1 (2.3)	2 (6.3)	0.391
Mental illness				
Major depressive episode	61 (81.3)	35 (81.4)	26 (81.3)	0.987
Generalized anxiety	64 (85.3)	36 (83.7)	28 (87.5)	0.647
Separation anxiety	28 (37.3)	21 (48.8)	7 (21.9)	0.017
Social phobia	50 (66.7)	28 (65.1)	22 (68.8)	0.741
Specific phobia	19 (25.3)	10 (23.3)	9 (28.1)	0.632
Attention-deficit/hyperactivity	27 (36.0)	27 (62.8)	-	-
Oppositional defiant	38 (50.7)	38 (88.4)	-	-
Conduct	20 (26.7)	20 (46.5)	-	-
**Parent/family characteristics**				
Age (years)	46.2 (6.7)	46.3 (6.9)	46.2 (6.6)	0.958
Female	65 (86.7)	37 (86.1)	28 (87.5)	0.855
Immigrant	10 (13.3)	7 (16.3)	3 (9.4)	0.413
Partnered	47 (62.7)	24 (55.8)	23 (71.9)	0.155
College/university graduate	50 (66.7)	31 (72.1)	19 (59.4)	0.248
Household income				0.309
<$30,000	10 (13.3)	6 (14.0)	4 (12.5)	
$30–$59,000	16 (21.3)	12 (27.9)	4 (12.5)	
$60–$89,000	21 (28.0)	8 (18.6)	13 (40.6)	
$90–$119,000	13 (17.3)	7 (16.3)	6 (18.8)	
$120–$149,000	7 (9.3)	5 (11.6)	2 (6.3)	
≥$150,000	8 (10.7)	5 (11.6)	3 (9.4)	
Psychological distress	66.2 (17.0)	65.1 (16.3)	67.7 (18.2)	0.519
**Geographic characteristics**				
Distance to hospital, ≥50 km	17 (22.7)	10 (23.3)	7 (21.9)	0.888
Residential instability				0.173
Q1 (lowest)	2 (2.7)	1 (2.3)	1 (3.1)	
Q2	21 (28.0)	10 (23.3)	11 (34.4)	
Q3	29 (38.7)	14 (32.6)	15 (46.9)	
Q4	15 (20.0)	11 (25.6)	4 (12.5)	
Q5 (highest)	8 (10.7)	7 (16.3)	1 (3.1)	

**Table 2:** Linear regression modeling assesses the association between internalizing–externalizing comorbidity and functioning.

	β	Standard Error	*p*
Internalizing–externalizing comorbidity	0.32	0.16	0.041
Child age, years	0.02	0.04	0.615
Child sex, female	0.08	0.17	0.649
Inpatient	−0.12	0.18	0.508
Household income	0.07	0.05	0.171
Parental psychological distress	0.01	0.01	0.004
Distance to hospital	0.38	0.19	0.049
Residential instability	−0.06	0.09	0.466

## Data Availability

Research data are not shared. Requests for data access can be made to the corresponding author.
